# Capsule Endoscopy in Patients with Cardiac Pacemakers and Implantable Cardioverter Defibrillators: (Re)evaluation of the Current State in Germany, Austria, and Switzerland 2010

**DOI:** 10.1155/2012/717408

**Published:** 2011-12-29

**Authors:** Dirk Bandorski, Ralf Jakobs, Martin Brück, Reinhard Hoeltgen, Marcus Wieczorek, Martin Keuchel

**Affiliations:** ^1^Medizinische Klinik 2, Universitätsklinikum Gießen, Klinikstraße 32, 35392 Gießen, Germany; ^2^Herz-/Kreislaufzentrum Rotenburg, Heinz-Meise-Straße 100, 36199 Rotenburg, Germany; ^3^Medizinische Klinik C, Klinikum Ludwigshafen, Bremserstraße 79, 67063 Ludwigshafen, Germany; ^4^Medizinische Klinik 1, Klinikum Wetzlar, Forsthausstraße 1, 35578 Wetzlar, Germany; ^5^Medizinische Klinik III, Herzzentrum Duisburg, Gerrickstraße 21, 47137 Duisburg, Germany; ^6^Klinik für Innere Medizin, Bethesda Krankenhaus Bergedorf, Glindersweg 80, 21029 Hamburg, Germany

## Abstract

*Background and Aims*. The study was a repeated evaluation of the experience of capsule endoscopy (CE) in patients with cardiac pacemaker or implantable cardioverter defibrillator (ICD). *Patients and Methods*. A standardized questionnaire was sent by the manufactors Given Imaging and Olympus to all centers in Germany, Austria, and Switzerland providing capsule endoscopy service. The questionnaire covers the number of examined patients, monitoring during CE, check of the electric implants before and after CE, occurrence of arrhythmia, quality of CE video, complications, indication of CE, and type of institution. *Results*. Overall 580 questionnaires were sent to the users. 26/5% (Germany/Austria + Switzerland) of the questionnaires were sent back anonymously to the authors. 114 centers (82 hospitals, 11 surgeries, 21 without specification) replied. In 58 centers (51%), patients with cardiac pacemaker (*n* = *300*) and ICDs (*n* = *80*) underwent uneventful capsule endoscopy. The predominant indication (patients with CP 97%, patients with ICD 100%) was mid gastrointestinal bleeding. *Conclusion*. The results of our inquiry show that in spite of formal contraindication CE is increasingly applied in bleeding patients with cardiac pacemakers/ICDs and seems to be safe even in a large cohort.

## 1. Introduction

During the last decade, capsule endoscopy (CE) has become an established tool for the investigation of the small intestine. However, the US Food and Drug Administration (FDA) as well as the manufactors Given Imaging and Olympus continue to recommend not using CE in patients with cardiac pacemakers and implantable cardioverter defibrillators (ICDs). Our in vitro investigations [1, 2] and several retrospective cohort studies [3, 4] did not reveal interference between capsule endoscopy and several types of pacemakers or ICDs, respectively. A survey in 2004 documented that CE had been applied in Germany in some patients with implanted cardiac devices in spite of formal contraindication and appeared to be safe [3]. In the meantime, two additional investigations for possible interference between CE and PMs or ICDs were published [5, 6]. This present survey reevaluates the clinical practice concerning CE in patients with pacemakers and ICDs in Germany. Additionally, centers in Austria and Switzerland were included. Furthermore, the influence of relevant publications on the use of CE in patients with pacemakers and ICDs is evaluated.

## 2. Methods

A standardized questionnaire was prepared and sent by Given Imaging and Olympus to all their customers running capsule endoscopy systems in hospitals and outpatient clinics in Germany (G), Austria (A), and Switzerland (S). The centers were asked to answer the 9 (A, S) or 10 questions (G), respectively, within 6 weeks anonymously. Corresponding to the survey in 2004 [3], the items polled included the number of CE performed in patients with pacemakers and ICDs, check of the devices before and after CE, monitoring during CE (ECG, intensive care unit), possible interference between CE and cardiac pacemakers/ICDs, observed arrhythmias during CE, adverse events during CE, indication for CE, the influence of publications on the application of CE in patients with pacemakers and ICDs, and finally country and type of institution (hospital/outpatient clinic). The questionnaire in Germany additionally evaluated the participation of the center in our survey of the year 2004.

## 3. Results

Given Imaging and Olympus sent a total of 580 questionnaires, 483 questionnaires in Germany (Given Imaging: 312 hospitals, 129 outpatient clinics; Olympus: 42 hospitals) and 97 questionnaires in Austria and Switzerland (Given Imaging: Austria 13 hospitals, Switzerland 39 hospitals, 24 outpatients clinics; Olympus: Austria 13 hospitals, Switzerland 7 hospitals, 1 outpatient clinics). The return of questionnaires amounted to 22.6% in Germany (109 of 483 questionnaires), in Austria and Switzerland to 5% (5 of 97 questionnaires). Fifty-eight centers (51%) used CE in patients with pacemakers or ICDs, 82 of them were hospitals (72%, G = 78, A/S = 4) and 11 outpatient clinics (10%, G). Twenty-one of the users did not reveal their type of institution (18%; G = 20, A/S = 1). In this survey, the number of reported CE patients with a pacemaker is 300 (Germany *n* = 286, Austria/Switzerland *n* = 14); the cumulative number of reported CE patients with an ICDs is 80 (Germany *n* = 75, Austria/Switzerland *n* = 5). Monitoring was done before CE in 16 patients (G), during CE in 29 (G = 28, A/S = 1), and after CE in 28 patients (G = 27, A/S = 1), respectively. In 8 clinics, patients underwent CE on the intensive care unit (ICU). Impairment of the quality of the CE video, artefacts or recording gaps of the video after installation of telemetry, was reported by 4 clinics. In two patients (supra-/ventricular) premature beats without clinical symptoms were observed. No complications were reported. Prevailing indication for CE was obscure GI bleeding (97% of the patients with a pacemaker, all patients with an ICD).

Twenty-six percent of the physicians reported that their decision to perform CE in patients with a pacemaker or an ICD had been positively influenced by relevant publications. No change in the approach to these patients was reported by 39% of the physicians, and 35% did not give further details to this question (Table 1). Three of the physicians (2.7%) had participated in our survey in the year 2004, 90 (86.6%) answered this question negatively, and 16 (14.7%) provided no information.

## 4. Discussion

Several studies and case reports about interference between pacemakers and ICDs have been published since our survey in the year 2004 [2, 4–9].

One group reported about interference with pacemakers and ICDs [10, 11]. For their in vivo and in vitro studies, a dedicated test cap (Given Imaging, Yoqneam, Israel) was used to simulate radio transmission of a PillCam. This test cap caused the pacemaker to revert to noise-mode function (VOO- or DOO-Mode) and provoked oversensing of ICDs. However, these findings could not be reproduced by others when using original capsule endoscopes in vitro and in vivo, and several case reports and series reported uneventful capsule endoscopy in an increasing number of patients with pacemakers or cardioverters. Furthermore, interference between CE (Given Imaging) and pacemakers seems to be impossible from a technical point of view (low emitted power of CE) even if CE and pacemakers/ICDs are in close proximity (personal communication by Professor Dr. Silny, head of research center for electromagnetic environment compatibility, RWTH Aachen, Germany, based on confidential technical data provided by Given Imaging). Summarizing the existing data, it may be concluded that CE seems to be safe even in the presence of implanted cardiac devices [12]. Correspondingly, 26% of the physicians reported in this survey that recent publications had reassured them not to withhold CE from these patients anymore, regardless of pacemakers and ICDs still being a formal contraindication.

A limitation of the present survey is the lack of information concerning the types and brands of the pacemakers and ICDs. However, increasing the time load to answer a more detailed questionnaire might have further decreased the low response rate. On the other hand, a detailed analysis of all different pacemaker and ICD device types in selected high-volume centers showed no clinically relevant interference between 19/8 types (pacemakers/ICDs) from 7 different brands and PillCam or Endocapsule systems [13].

In this survey, indication for CE was almost exclusively obscure gastrointestinal bleeding. This is similar to the results of the 2004 survey but different from other series on caspule endoscopy in unselected patients, where GI bleeding accounted for approximately 66% of indications [14]. Although not included in the questionnaire, it might be suspected that patients with implanted cardiac devices might be older and more frequently suffer from comorbidity and require anticoagulants and thrombocyte aggregation inhibitors, thus provoking GI bleeding.

Although CE does not seem to influence cardiac devices in clinical practice, four clinics report about interference of CE (artefacts, stopping of recording of the video) after instillation of telemetry. In a retrospective multicenter investigation, interference (artefacts, impossibility to document CE images) between CE and telemetry occurred in two cases [13]. Reasons for this interference are disturbances on the same frequency as CE. Many wireless applications use the frequency of 434 MHz (transmission range of CE). Remarkably these interferences do not occur permanently. Disturbances between CE and telemetry could possibly explain the interferences with impairment of the CE video in the studies of Guyomar et al. [15] and Bandorski et al. [3].

Monitoring or tests of the implanted devices before and after CE were performed only by half of the physicians. Although this fact limits the power of the present study to detect asymptomatic arrhythmias, it demonstrates physicians' confidence into the safety of CE, thus avoiding presumably unnecessary precautions or potential disturbance of CE videos.

Despite of the low response rate in this survey, the number of included patients with pacemakers and ICDs who underwent CE increased to 380 compared to 53 in our last survey in the year 2004. As only three physicians stated in the 2010 survey that they had participated in the 2004 inquiry (where 28 physicians had performed CE in patients with cardiac devices), the total number of patients might further be underestimated.

In spite of limited details provided by the present survey, this largest cohort of patients with a pacemakers or implanted cardioverter undergoing an uneventful capsule endoscopy adds further evidence to support its safety.

## Figures and Tables

**Table 1:** Results.

	Number of hospitals/outpatient clinics (*n* = 58)
Control of cardiac device before CE	
Yes	16 (G)
No	34 (G =33, A/S = 1)
No reply to this question	8 (G = 7, A/S = 1)
Control of cardiac device after CE	
Yes	28 (G = 27, A/S = 1)
No	27 (G = 26, A/S = 1)
No reply to this question	3 (G)
ECG during CE	
Yes	29 (G = 28, A/S = 1)
No	24 (G)
No reply to this question	5 (G = 4,A/S = 1)
CE on ICU	
Yes	8 (G = 7, A/S = 1)
No	47 (G)
No reply to this question	3 (G = 2, A/S = 1)
Disturbance of video	
Yes	4 (G)
No	54 (G = 52, A/S = 2)
Interference of pacemaker/ICD	
No	58
Arrhythmias	
Yes	2 (G; SVES, VES)
no	56 (G = 54, A/S = 2)
Other complications	
Yes	0
No	57 (G = 56, A/S = 1)
No reply to this question	1 (A/S)
Indication of CE	
Bleeding	54 (G = 52, A/S = 2)
Inflammatory bowel disease	1 (G)
Other	2 (G)
No reply to this question	1 (G)

All (with und without application of CE, *n* = 114)	

Type of institution (all G : 109, A/S : 5)	
Hospital	82 (G = 78, A/S = 4)
Outpatient clinic	11 (G)
No reply to this question	21 (G = 20, A/S = 1)
Modification of application of CE	
Yes	30 (G)
No	44 (G = 43, A/S = 1)
No reply to this question	40 (G = 36, A/S = 4)

CE: capsule endoscopy, ECG: electrocardiogram, ICU: intensive care unit, G: Germany, A: Austria, S: Switzerland.
